# Temporary intravascular shunts and limb salvage in civilian vascular trauma

**DOI:** 10.3389/fsurg.2023.1302976

**Published:** 2023-11-24

**Authors:** Ombretta Martinelli, Francesca Miceli, Simone Cuozzo, Francesco Giosuè Irace, Stefano Avenia, Immacolata Iannone, Ilaria Clementi, Paolo Sapienza, Maria Irene Bellini

**Affiliations:** ^1^ Department of General and Specialty Surgery, Sapienza University of Rome, Rome, Italy; ^2^ Department of Surgery, Sapienza University of Rome, Rome, Italy

**Keywords:** trauma, limb salvage, amputation, vascular injury, vascular shunt

## Abstract

**Background:**

Temporary intravascular shunts (TIVS) may allow quick revascularization and distal reperfusion, reducing the ischemic time (IT) when an arterial injury occurs. Furthermore, TIVS temporarily restore peripheral perfusion during the treatment of concomitant life-threatening injuries or when patients require evacuation to a higher level of care. Notwithstanding, there are still disputes regarding the use of TIVS, in view of the paucity of evidence in terms of potential benefits and with regard to the anticoagulation during the procedure. The present study aimed to assess TIVS impact, safety, and timing on limb salvage in complex civilian vascular traumas.

**Patients and methods:**

Data were retrieved from the prospective database of our department, which included all patients hospitalized with a vascular injury of the extremities between January 2006 and December 2022. Patients undergoing TIVS during vascular injury management were included in group A, and those who could not postpone immediate care for TIVS insertion were included in group B (control group). Data concerning the times required for extremity revascularization or other surgical procedures such as orthopedic interventions and the time of limb ischemia were compared between the two groups. A comparison of the postoperative course between the two groups was also performed.

**Results:**

A total of 53 patients were included: group A (TIVS insertion, *n *= 31) and group B (control, *n *= 22). Revascularization time significantly differed (*p *= 0.002) between the two groups, which is lower in group A (4.17 ± 2.37 h vs. 5.81 ± 1.26 h). TIVS positively affected the probability of limb salvage (*p *= 0.02). At multivariate analysis, the factors independently associated with limb salvage were TIVS usage, the necessity of hyperbaric oxygen therapy, and the total IT. In group A, there were three deaths and one major amputation, and in group B, there were two deaths and four major amputations.

**Conclusions:**

The use of TIVS minimizes revascularization time and improves limb salvage probability. A multidisciplinary approach is recommended, and correct surgical timing is key to ensure the best outcome.

## Introduction

The management of complex vascular trauma of the lower limbs continues to pose several questions regarding diagnosis and treatment, with the risks of bleeding, ischemia, and even death (1, 2). Despite surgical intervention, the amputation rate remains between 14% and 69% (3, 4).

This high incidence of amputation is mainly related to the time between the onset of the ischemic event and the revascularization time, although other factors may contribute, such as the nature of the injurious agent itself, the presence of bone fractures, and the extent of tissue lesions.

Although the estimated ischemic time (IT) after which recovery and functional outcome would not occur is > 6 h, in patients with complex or multiple traumas, hemodynamic shock, direct vascular injuries, loss of collateral circulation, and the associated venous lesions could synergistically affect the tolerance to the IT, resulting in severe damage of muscular and nervous tissues (5).

Furthermore, these situations might promote the onset of compartment and/or revascularization syndrome with related mortality rates of 4%–5% (6).

It is understandable why the arterial flow should be restored as quickly as possible to save a limb with optimal functionality, and the use of temporary intravascular shunts (TIVS) may play a key role in increasing the limb salvage rate (7).

In this area, the use of temporary arterial and venous shunting has been advocated to obtain better management and rapid recovery of distal perfusion (8, 9).

Several advantages can be obtained with TIVS before bone fracture stabilization (8); in fact, they represent a way to allow orthopedic fixation without worsening the vascular ischemia and also preventing systemic effects related to the absorption of the released toxic ischemic products (8).

Moreover, TIVS can minimize the need to perform fasciotomy, determining a net improvement in terms of limb salvage. Notwithstanding, there are still disputes regarding the use of TIVS, due to the scarcity of literature on its real benefit and in relation to the need for anticoagulation during shunting.

In addition, the indication of shunting is still controversial, since its use may be difficult because of the injury location (proximal vs. distal) and severity, the time to presentation, the shunt type, and the expertise of the surgeon.

The present study aims to assess the impact of TIVS on limb salvage in complex vascular trauma of lower limbs.

## Patient and methods

We included all consecutive patients with multiple trauma and complex vascular injuries of the lower limbs who were treated using TIVS in the Vascular Surgery Unit and Trauma Centre at Umberto I Hospital, Rome, Italy, between January 2006 and June 2018. The inclusion criteria were a vascular injury to the common, superficial, or deep femoral arteries. The patients with incomplete data were excluded. In case of multiple vascular injuries, the analysis has concerned the damaged vessel in which a shunt was inserted or could have been inserted.

The patients were grouped according to the use of shunting of the injured vessel: group A with TIVS and group B without TIVS. TIVS insertion decision was assessed if there was no contraindication to postpone surgical treatment; if this was not the case, injured patients went to the theater without TIVS.

The diagnosis was performed by a multidetector computed tomography angiography (MDCTA) at the time of presentation for the initial assessment of the vascular injury, as per the standard of care in the diagnosis and characterization of patients with vascular trauma (10).

The surgical phases of the intervention were as follows:

(1) surgical vessel preparation and insertion of shunts,
(2) bone stump realignment and external fixator positioning,
(3) vascular reconstruction,
(4) fasciotomy, and
(5) intraoperative angiographic control.

The decision to insert the shunt was made on a case-by-case basis at the moment of surgery. TIVS were inserted for the injuries of the femoral vessels, in relation to the small caliber of the distal injured arteries and the inherent greater risk of shunt thrombosis.

The main indications for TIVS were systemic hemodynamic instability (systolic blood pressure < 90 mmHg), IT before surgery > 6 h, severe acute ischemia, and simultaneous orthopedic surgery. In the latter cases, the shunt was inserted at the start of the procedure, and definitive repair was carried out once the fracture was fixed.

No systemic anticoagulation was administered. Local intra-arterial heparinized saline was routinely given. When IT exceeded 6–8 h, a “washing of the limb” with heparinized saline and Ringer lactate was performed, with a remnant 200–400 cc venous blood flow. If the time to reperfusion exceeded 4–6 h or if there were clinical signs of tense compartments after reperfusion, fasciotomy was performed.

Vacuum-assisted closure (VAC) and hyperbaric oxygen therapies were used when needed. At the end of the revascularization, a completion angiography was carried out in patients diagnosed with complex vascular injury (10).

Follow-up controls included clinical evaluation, duplex scanning before discharge and at 3–6 and 12 months, and MDCTA at 1 month after surgery.

### Statistical analysis

Data concerning the times required for limb revascularization comprehensive of other surgical procedures such as orthopedic interventions and the times of limb ischemia were compared between the two groups. Data on the postoperative course of the patients in the two groups were also analyzed.

The categorical variables were reported as frequencies, and the continuous variables were reported as mean ± standard deviation with range. Comparisons between groups were performed using the chi-square test for categorical variables and *t*-tests for continuous variables. Kaplan–Meier survival estimates and a multivariate analysis of the included variables assessed factors related to limb salvage. SPSS version 27 was used for the analysis. A *p*-value of less than 0.05 was taken for statistical significance.

## Results

A total of 53 patients were included in the analysis (30 men, aged between 23 and 67 years, mean 36.5 years ± 9.8), and all were admitted to our unit and presented with multiple trauma and complex vascular lesions of lower limbs, caused by blunt (*n *= 44) or penetrating traumas (*n *= 9).

The type and site of the arterial trauma, the concomitant injuries, and the additional procedure in the two groups are presented in Table 1.

**Table 1:** Results.

		Group A TIVS	Group B control	*p*
Trauma	Blunt	26	18	ns
	Penetrating	5	4	
Arterial injury	Femoral	22	15	0.042
	Popliteal	5	6	
	Distal	4	1	
Concomitant injury	None	17	7	ns
	Vein	9	7	
	Nerve	1	6	
	Vein + nerve	4	2	
Injury in other sites	Spleen	10	6	ns
	Aortic arch	1	0	
	Pelvic Fracture	6	2	
	None	2	7	
	Head	9	4	
	Upper limb	3	3	
Bone lesion	No	12	8	ns
	Yes	19	14	
Fasciotomy	No	14	2	0.004
	Yes	17	20	
Hyperbaric	No	20	15	0.038
	Yes	11	7	
Arterial open repair	Open	31	22	ns
Graft thrombosis		0	2	ns
Additional procedure	None	25	8	0.007
	Nerve reconstruction	1	5	
	Muscle/skin reconstruction	4	8	
	Muscle flap	1	1	

Damage to the femoral artery was present in 37 patients, to the popliteal in 11 patients, and to the infragenicular arteries in five patients.

Other concomitant traumatic lesions occurred in 44 patients: spleen (*n *= 16), head trauma (*n *= 13), upper limbs fractures (*n *= 6), pelvis fractures (*n *= 8), and aortic isthmus rupture (*n* = 1).

The diagnosis of the vascular injury was clinical in four patients, who were admitted to our center while in hemorrhagic shock and immediately operated on.

The vascular trauma of the lower limbs was diagnosed by CT in 48 patients and by color duplex scanning (CDS) in the remaining cases. In four patients, the arterial injury was confirmed by the intraoperative angiography during the endovascular treatment.

All patients with splenic rupture were first submitted to splenectomy.

Among the 13 patients with head injury, only two patients were treated with craniotomy and hematoma evacuation before vascular lesion treatment.

The patient with aortic isthmus rupture was submitted to an endovascular treatment as the first procedure.

Surgical repair of the arterial lesion of the limbs was performed between 1.3 and 8.17 h (average 5.1 ± 1.95 h) from the trauma.

Group A (with TIVS) included 31 patients, and group B (control) included 22 patients. In group A, the average injury severity score (ISS) was 17 points, and only 13.5% had an ISS of 30 or greater. In group B, the average ISS was 18 points, and only 14% had an ISS of 30 or greater. In group A, a Sundt® NeuroCare® 3.5-mm shunt (Integra, Plainsboro, NJ, USA) was used in five patients, whereas Pruitt-Inahara® shunt (LeMaitre Vascular Inc., Burlington, MA, USA) was employed in 26 patients. Concomitant venous lesions were present in 16 patients (nine in group A and seven in group B), nerve lesions in seven patients (three in group A and four in group B), and associated veins + nerves in six patients (two in group A and four in group B). The anatomic location of the arterial femoral trauma was significantly more frequent in the shunted group (*p* = 0.042), while the injury nature did not differ between the two groups and the length of the arterial lesion: 5.8 cm (1–13 cm) vs. 5.6 cm (1–12 cm) in groups A and B, respectively.

In both groups, IT prolongation due to the orthopedic intervention was between 1 and 15 h, with a mean of 5.43 ± 2.88 h.

As a first step, vein reconstruction was carried out when vein injuries occurred in conjunction with arterial damage in nine patients in group A and seven patients in group B.

The types of arterial repair performed with TIVS and control groups are listed in Table 2.

**Table 2:** Types of open arterial repair.

	Group A (%)	Group B (%)	*p*
End-to-end anastomosis	9 (29.1)	4 (18.2)	ns
Autologous saphenous vein interposition or bypass graft	13 (41.9)	5 (22.7)	ns
Synthetic graft interpositions or bypass	5 (16.21)	8 (36.4)	ns
Artery repair with a saphenous vein or Dacron patch	2 (6.4)	1 (4.6)	ns
Ligation of arterial injury	2 (6.4)	4 (18.2)	ns

ns, not significant.

An end-to-end anastomosis repair was feasible to repair a retrogeniculate popliteal artery trauma in eight patients (five in group A and three in group B). All the open and endovascular interventions were carried out by vascular surgeons.

All vascular shunts maintained their patency throughout their entire dwell time. The patency of the shunt was confirmed by intraoperative CDS.

At the end of the revascularization, a completion angiography was carried out.

Five patients died during the postoperative period: disseminated intravascular coagulation in three patients and cerebral hemorrhage in two patients.

Concerning the remaining 48 patients, five patients were submitted to a major amputation, due to vein graft occlusion (*n *= 2) (two patients without TIVS insertion) and infection (*n *= 3). In the two patients, there was an occlusion of the graft caused by the poor distal runoff following thrombosis of two out of three below-knee arteries: proximal arterial trauma in one case and severe persistent systemic hypotension in the other. Sciatic nerve injury was present in a couple of them (*n *= 2).

In the remaining patients (*n *= 43), limb salvage was obtained, and no postoperative complications related to revascularization were observed.

In the case with associated head trauma (*n *= 1), a right hemiplegia with partial recovery occurred.

To summarize, according to the use of TIVS, we observed:

1. Group A: TIVS insertion: three died in the postoperative period and one underwent major amputation.
2. Group B: without TIVS: two died and four major amputations were performed.

**Figure 1:**
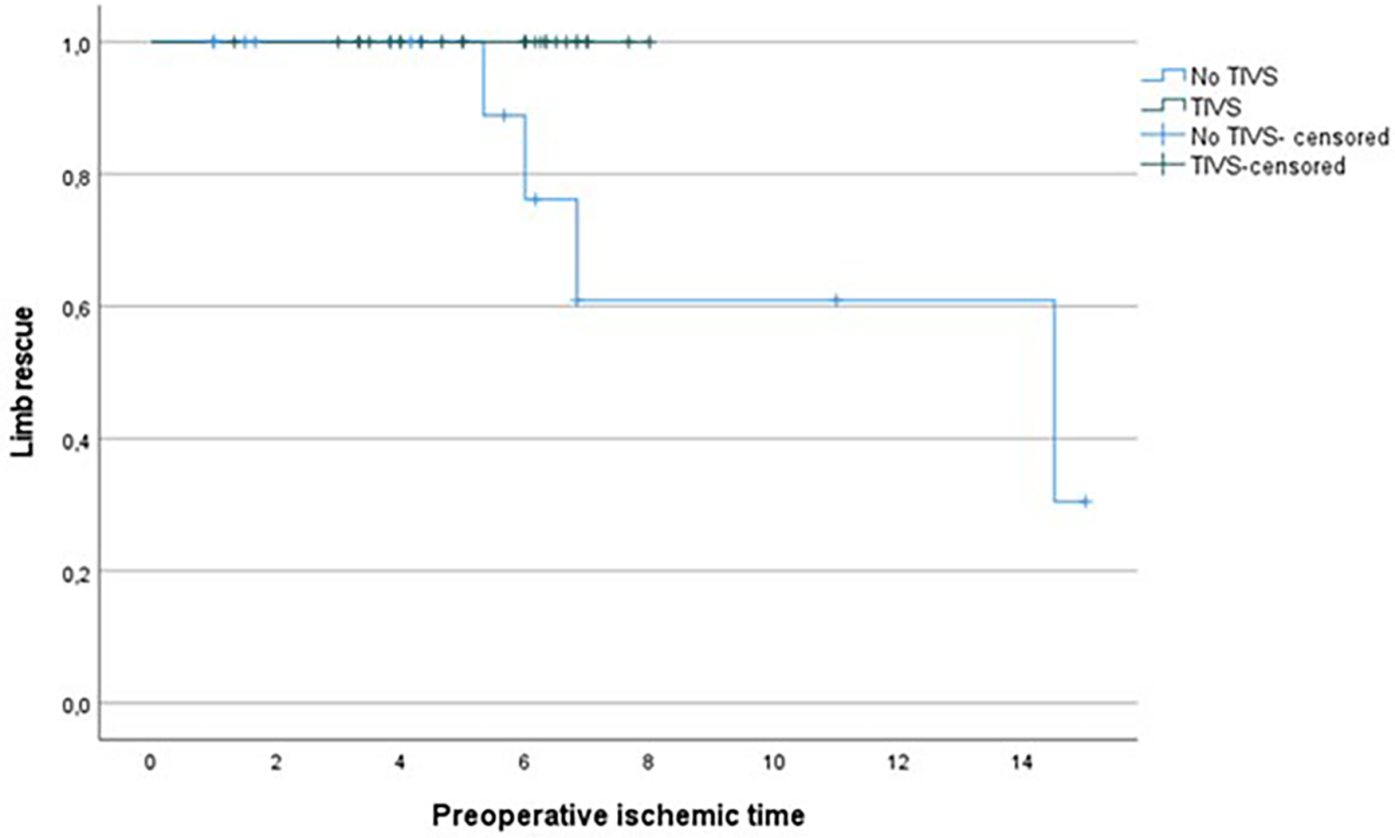
Kaplan Meier for limb rescue probability by total ischemic time in TIVS and non-TIVS groups. Log Rank 0.014.

**Figure 2:**
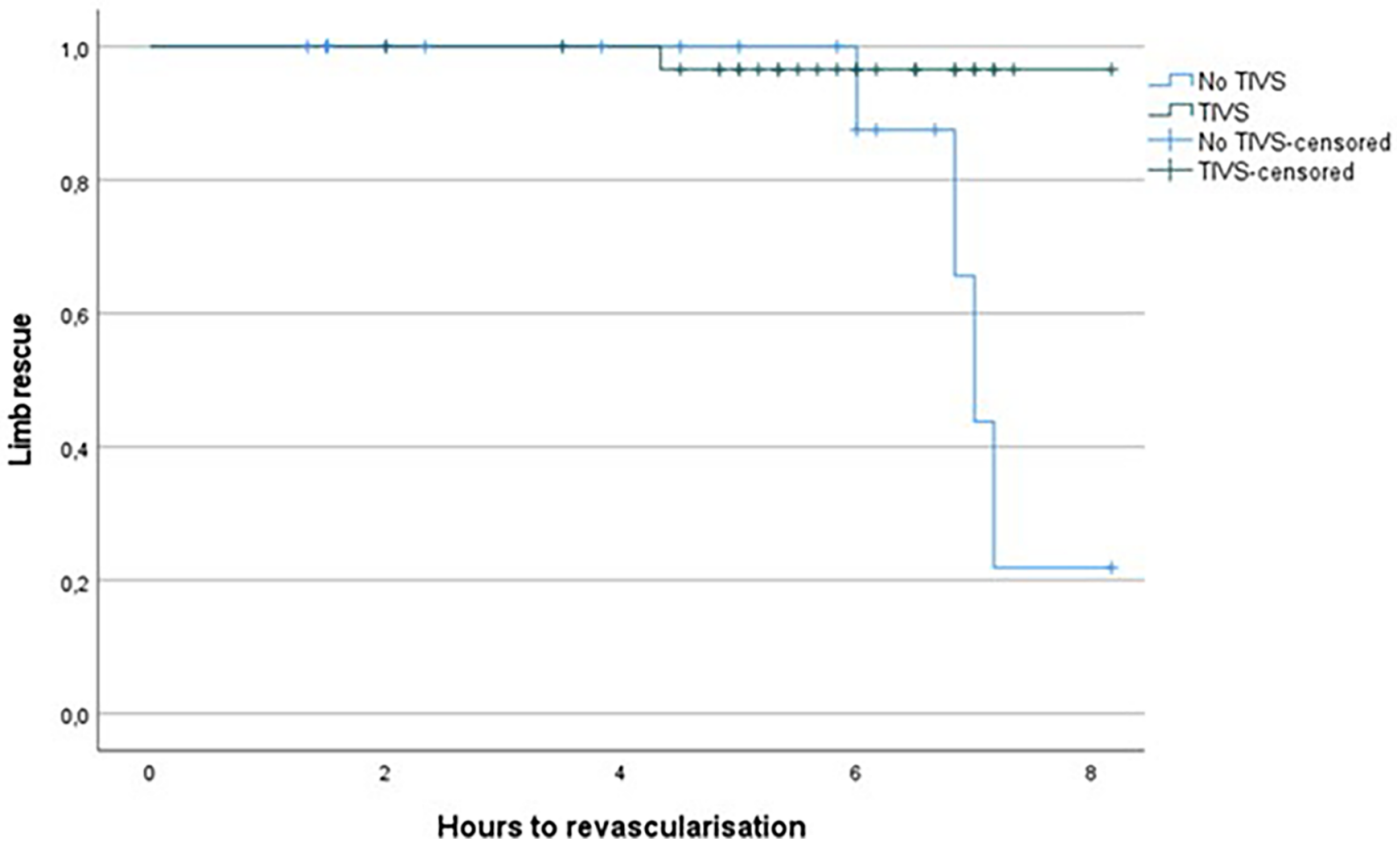
Kaplan Meier for limb rescue probability by hours prior to revascularization in TIVS and non-TIVS groups. Log Rank 0.02.

Revascularization time significantly differed (*p *= 0.002) between the two groups, with lower time in the TIVS group, 4.17 ± 2.37 h vs. 5.81 ± 1.26 h (Figure 1). TIVS positively affected the probability of limb salvage (*p *= 0.02) (Figure 2). At multivariate analysis, the factors independently associated with limb salvage were as follows: TIVS usage, the necessity of hyperbaric oxygen therapy, and the time of orthopedic intervention (Table 3).

**Table 3:** Multivariate analysis of factors independently associated with limb salvage.

Variable	*p*
Age	0.704
Type of trauma	0.367
Site	0.210
Venous lesion	0.215
Other lesion	0.953
Bone lesion	0.086
Time to revascularization	0.113
TIVS	**0.008**
Time of orthopedic intervention	**0.047**
Fasciotomy	0.152
Hyperbaric oxygen therapy	**0.005**
Type of arterial repair	0.544
Additional procedure	**0.061**

Bold values are statistically significant.

## Discussion

IT is the main factor influencing the outcome of vascular injuries of the lower limbs in terms of amputation rates and functional recovery, as observed by Sanderson et al. (11) who indicated that myonecrosis starts after 6–8 h of ischemia and the amputation rate is equal to 86%. Moreover, ischemia combined with direct muscular damage produces a cellular edema within 2–3 h and increases the muscle volume and the consequent establishment of a compartment syndrome (12). The severity and duration of ischemia following a vascular trauma are related to the injury location and time of presentation. It becomes evident then the importance of a rapid reperfusion of the limb.

As reported in fact by Smith et al. (13), Rich and Sullivan (14), and McNamara et al. (15), complex associated bone fractures, venous lesions, and loss of muscle tissue and skin contribute to the unfavorable outcomes of limb salvage (16).

In case of hemorrhagic shock, preoperative investigations are not possible, so after bleeding control, an intraoperative angiography is advised and in many cases is the best diagnostic tool. In other cases, a total body scan could be successful in detecting bone, vascular, and associated cranial, thoracic, and abdominal lesions (17). CDS evaluation can be quickly performed to evaluate distal perfusion of tibial and pedal arteries (18).

Data from the present analysis have shown that TIVS use provides quick and effective extremity perfusion in the most severely injured limbs. The results from the present study have confirmed that TIVS minimize the total IT throughout bone fixation, vascular reconstruction, and nerve reparation (19). In addition, shunting results in a washing limb, a loco regional heparinization, and a venous outflow, with muscle edema reduction. Our results are consistent with the data reported by Inaba et al. (20) who showed that shunted patients had reduced IT, fasciotomy and amputation rates, and repeat operations compared to the non-shunted group.

According to our experience, systemic heparinization is not advisable, as in most cases polytrauma is present with multiple organ involvement and the consequences of uncontrolled bleeding could not always be controlled.

From our data, the arterial shunt eventually combined with vein shunting, allows a rapid reperfusion of the ischemic tissue in severely mangled extremities improving limb salvage, neuromuscular recovery, and functional outcome with good shunt patency and no increased risk of graft thrombosis. This seems to disprove the hypothesis that shunts may cause endothelial injury and subsequent vascular graft thrombosis (21).

Although there is no proven association between the use of different types of shunts and subsequent graft failure, our results may be related to the size and type of the shunts inserted in the injured vessels.

In our experience, the Sundt® shunt was employed in five patients, whereas the Pruitt-Inahara® shunt was employed in 26 patients: these types of shunts with a diameter of 3.5–3 mm reintroduce an adequate flow with sufficient limb perfusion if systemic pressure is about 80 mmHg. The Sundt® shunt usually restores greater flow, whereas the Pruitt-Inahara® shunt is easier to implant and provides a way for distal heparin infusion.

The first evidence to demonstrate TIVS superiority to ligation to minimize consequences of vascular trauma ischemia was published by Khalil and Livingston (22) in 1986. There are multiple advantages: beyond allowing bone stabilization, with limb temporary reperfusion, it permits to carry out any other priority intervention, for example, for spleen rupture, thoracic blunt trauma, or chronic trauma as reported in the literature (23–26). In our experience, the patients were less likely to have undergone arterial injury ligation (Table 2) since restoration of blood flow through temporary shunting minimized the IT to less than 6 h to allow arterial reconstruction for maximum limb salvage. Although limited by a small cohort of a single center with its inherent bias, our experience shows a decrease in amputation rate from 62.5% without shunting to 23.6% with TIVS usage (*p *= 0.008) in an acute setting and a reduction of graft thrombosis from 25% to 0% in matched controls, which is attributable to the lower impairment of the distal runoff secondary to shunting. Similarly, Subramanian et al. (27) reported three graft thromboses out of 35 cases of the largest previous civilian series of the use of TIVS in the vascular injury control setting.

Regardless of the mortality rates due to the severity of the concomitant injury traumas, we think that TIVS patients could benefit from this particular treatment also because of a reduction in septic complications caused by ischemia itself, as well as a minor incidence of postoperative graft occlusion. Infection represents a very dangerous complication in many patients, hardly controllable with antibiotic therapy, and may increase the rate of amputations. Temporary arterial shunting may decrease the rate of infection since it provides more time to perform an arterial reconstruction with a autologous saphenous vein graft and reduces the need for fasciotomy. In addition, the routine use of VAC and hyperbaric therapy appears to have reduced infectious complications, increasing the limb salvage rate in our series.

These data confirm the evidence of Oliver et al. (21) showing that a temporary shunt can be a life- and limb-saving option.

TIVS indication must be weighted with respect to IT severity and duration with respect to the risk of its complications. From the present study, for IT ≤6 h, TIVS does not change, in a statistically significant way, the outcomes in terms of limb salvage; conversely, when IT is ≥6 h, TIVS use turns out to be significantly advantageous.

Some authors do not recommend inserting the shunt in the vein due to the possibility of wall lesions and venous thrombosis (24, 28). Moreover, in many cases, the vein is irreversibly damaged, and its ligation is the best option. In the latter situation, the use of a contralateral saphenous vein for arterial repair should be recommended (29–32). Anyhow, there is evidence that vein shunting and repair is associated with lower incidences of compartment syndromes, fasciotomies, and amputations (32).

Finally, despite tourniquets are thought to reduce intraoperative bleeding and improve the view in the surgical field, we do not routinely use them, because of the potential increase in venous thromboembolism, neurovascular injury, wound complications, including infection, and higher rates of limb loss following local tissue hypoxia (33). In our series, direct pressure was an adequate life-saving anti-exsanguination modality, and the tourniquet was never used. In the present study, TIVS placement allows venous reconstruction when feasible, which must precede the arterial one, and a graft interposition is preferable by end-to-end anastomosis (34).

Regardless of whether to use the shunt or not, a close interdisciplinary coordination of the various surgical procedures is, however, imperative to guarantee an optimized stable reconstructive outcome with acceptable patient risk.

As shown from our study, the treatment of complex injuries of the lower limbs may require differentiated and interdisciplinary surgical approaches, since in some cases, accompanying soft tissue defects had to be reconstructed after wound debridement by plastic surgeons especially when myocutaneous flaps were needed.

Timely and definitive treatment of complex fractures should be carried out by the orthopedic team as promptly as possible to reduce the immobilization periods.

In patients with complex and severe trauma, nerve injuries determine the functional recovery of the limb (25). In many cases, it is impossible to precisely detect the entity of neurologic status. Whenever possible, a reconstruction of the injured nerve, by the neurosurgery team, is advised. In some instances, the autologous sural nerve can be employed with discrete results as also observed in our series (35).

## Conclusions

Temporary intravascular shunt placement may play a role in preserving limb viability in civilian trauma to control hemorrhage and rapidly reestablish flow reducing IT. With regard to vascular reconstruction, temporary shunting in damage control situations allows the most effective vessel revascularization and the use of autologous prosthetic material when available. Notwithstanding, given the number of complicating factors of vascular traumas in terms of severity, time to presentation, and site of the trauma, the indications for shunt use should be the result of a multidisciplinary approach by a managing team including an orthopedic surgeon, a vascular surgeon, a radiologist, a plastic surgeon, and a neurosurgeon. It follows that the choice cannot be generalized but evaluated for each patient to achieve the best management of complex vascular trauma of limbs.

## Data Availability

The original contributions presented in the study are included in the article/Supplementary Material, further inquiries can be directed to the corresponding author.
